# Transboundary Spread of *Brucella canis* through Import of Infected Dogs, the Netherlands, November 2016–December 2018

**DOI:** 10.3201/eid2707.201238

**Published:** 2021-07

**Authors:** Marloes A.M. van Dijk, Marc Y. Engelsma, Vanessa X.N. Visser, Ingrid Keur, Marjolijn E. Holtslag, Nicole Willems, Björn P. Meij, Peter T.J. Willemsen, Jaap A. Wagenaar, Hendrik I.J. Roest, Els M. Broens

**Affiliations:** Utrecht University Faculty of Veterinary Medicine, Utrecht, the Netherlands (M.A.M. van Dijk, N. Willems, B.P. Meij, J.A. Wagenaar,^,^ E.M. Broens);; Wageningen Bioveterinary Research, Lelystad, the Netherlands (M.Y. Engelsma, M.E. Holtslag, P.T.J. Willemsen, J.A. Wagenaar, H.I.J. Roest);; Netherlands Food and Consumer Product Safety Authority, Utrecht (V.X.N. Visser, I. Keur);; Ministry of Agriculture, Nature and Food Quality, The Hague, the Netherlands (H.I.J. Roest)

**Keywords:** Canine brucellosis, *Brucella canis*, zoonoses, bacteria, the Netherlands

## Abstract

*Brucella canis* had not been isolated in the Netherlands until November 2016, when it was isolated from a dog imported from Romania. Including this case, 16 suspected cases were notified to the authorities during the following 25 months. Of these 16 dogs, 10 were seropositive; tracking investigations found another 8 seropositive littermates. All seropositive animals were rescue dogs imported from Eastern Europe. *B. canis* was cultured from urine, blood, and other specimens collected from the dogs. Genotyping of isolates revealed clustering by litter and country. Isolating *B. canis* in urine indicates that shedding should be considered when assessing the risk for zoonotic transmission. This case series proves introduction of *B. canis* into a country to which it is not endemic through import of infected dogs from *B. canis*–endemic areas, posing a threat to the naive autochthonous dog population and humans.

Canine brucellosis is caused by the bacterium *Brucella canis.* Reproductive disorders such as late abortion, stillbirth, epididymitis, and sperm anomalies are most frequently observed (*1*). Other clinical signs are lymphadenitis (*1*,*2*) and musculoskeletal disease (e.g., discospondylitis) (*3*). In addition, the infection can remain subclinical (*2*). *B. canis* is mostly transmitted vertically from bitch to offspring or venereally through vaginal discharge and semen; urine has also been implicated as a possible mode of transmission (*1*,*4*,*5*).

*B. canis* is a zoonotic pathogen; humans can become infected through direct contact with secreta and excreta of infected dogs (*6*,*7*) or through laboratory exposure (*8*,*9*). Clinical signs in humans vary from subclinical infection (*10*) to fever, malaise, splenomegaly, and lymphadenopathy (*7*). Human cases of *B. canis* infection are reported infrequently. However, the prevalence of human *B. canis* infections is probably underestimated; the diagnosis might be missed because of nonspecific clinical signs and the absence of accurate serologic tests for *B. canis* antibodies in humans (*6*,*11*). In the United States, a seroprevalence of 3.6% was found among persons occupationally exposed to dogs. Two seropositive persons had clinical symptoms of brucellosis, and both reported contact with *B. canis*–seropositive dogs (*10*). In addition, an outbreak involving 6 seropositive persons, 5 of whom had clinical symptoms, was described after contact with a seropositive litter (*6*). In general, *B. canis* appears to cause less severe clinical symptoms in humans than other *Brucella* spp. (*12*). However, the public health relevance of *B. canis* needs further investigation before a proper risk assessment can be performed.

*B. canis* is considered endemic in the southern United States, Central America, and South America and has been reported from Canada, Asia, Africa, and Europe (*7*,*13*). Sporadic cases originating from northwestern Europe have been reported and were at least partially caused by importing an infected dog (*13*,*14*). Recent papers have expressed concerns about the introduction of *B. canis* in countries to which it is not endemic through infected dogs (*15*,*16*). Brucellosis in dogs is, in contrast to livestock, not notifiable to the World Organisation for Animal Health (OIE) or the European Union (EU directive 64/432/EEG). In the Netherlands, brucellosis is notifiable in humans and all mammal species (*17*,*18*). *B. canis* had not been isolated in the Netherlands until November 2016, when it was isolated from a dog imported from Romania that had discospondylitis. Raised awareness following this first case resulted in multiple notifications at the Incidence Crisis Centre (NVIC) of the Netherlands Food and Consumer Product Safety Authority (NVWA). This study describes the follow-up of these notifications and the implications for animal and human health.

## Methods

### Notifications and Study Period

Animal owners, veterinarians, and laboratories in the Netherlands are obliged to notify suspicions of brucellosis to the competent authority, the NVWA, according to Dutch legislation (*17*,*18*). Suspicions are mostly based on clinical signs compatible with brucellosis and a history of importation. In this study, we include all notified and related *B. canis* cases during November 2016–December 2018, provided there was a clinical suspicion (e.g., routine tests for export or import excluded), and diagnostic tests were performed at the National Reference Laboratory (NRL; Wageningen Bioveterinary Research, Lelystad, the Netherlands). No mandatory control measures for pets are in place once a positive case has been identified.

### Tracking Investigations

Upon notification, NVIC began investigations to track potential transmission by taking samples from suspected dogs and (if applicable) contact dogs or littermates for serologic and bacteriologic (blood and urine) evaluation. Contact dogs were defined as any dog imported with, cohabiting with, or regularly spending time with the suspected dog. Dogs were considered positive if they tested positive for *B. canis* antibodies or when the bacterium was cultured from blood, urine, or infection sites. In case of euthanasia of a seropositive dog, postmortem examination was performed by the NRL, and samples of various tissues were collected for culture. Diagnostic tests were performed by the NRL.

### Detection of *B. canis* Antibodies

Serum samples were tested for *B. canis*–specific antibodies by the 2-Mercapto-ethanol serum agglutination test as described by Alton et al. (*19*) as reference method with an in-house derived positive rabbit anti–*B. canis* control serum (NRL in-house validation). Interpretation of the antibody titer is ˂1:50 negative, 1:50–1:100 inconclusive, >1:200 positive (*19*).

### Detection of *B. canis*

#### Culture

We isolated *Brucella* spp. from clinical and tissue samples according to the OIE protocol (*20*). All laboratory work with potential *Brucella*-contaminated samples was performed within a Biosafety Level (BSL) 3 facility. Suspected colonies were confirmed as *Brucella* spp. by matrix-assisted laser desorption/ionization time-of-flight mass spectrometry on the Bruker MALDI Biotyper (Bruker, https://www.bruker.com) by using an extended in-house *Brucella* spp. database (*21*) and PCR.

### DNA Isolation, PCR, and Genotyping

DNA from tissue samples was extracted by using the DNeasy Blood and Tissue Kit (QIAGEN, https://www.qiagen.com). DNA isolation from *Brucella*-suspected colonies was performed by suspending the colony in 200 μL nuclease-free water (Sigma-Aldrich, https://www.sigmaaldrich.com) and boiling at 100°C for 8 min, followed by centrifugation for 2 min at 20,000 × *g*. We performed real-time PCR targeting the IS711 sequences of *Brucella* spp. (*22*). Colonies and tissue samples were considered positive after real-time PCR if the results showed a cycle threshold (C_t_) value of <36 (with sigmoid curve), inconclusive if C_t_ value was >36 but <40 (with inconclusive sigmoid curve), and negative if C_t_ value was >40 or there was no C_t_ at all.

For in silico multiple-locus variable number tandem repeat analysis (MLVA) and multilocus sequence typing (MLST), we constructed fragmented libraries by using Nextera DNA sample preparation kit (Illumina, https://www.illumina.com), as earlier published (*21*). Next generation whole-genome sequencing was performed by paired-end sequencing (300-bp reads) by using the Illumina technology on the MiSeq instrument (Illumina). We performed de novo assembly of the quality filtered reads by using ABySS-pe version 1.3.3 (*23*). Reads were aligned by using Bowtie2 version 0.2 (http://bowtie-bio.sourceforge.net/bowtie2/index.shtml) to the assembled contigs and the contig sequences were manually verified by using Tablet version 14.04.10 (*24*). We performed in silico MLVA-16 clustering according to the algorithm as described previously (*25*) by using Bionumerics version 7.6 (Applied Maths, https://www.applied-maths.com) and assigning MLVA-type from DNA-sequence with software (*26*) or manually. MLST typing was performed in silico with a set of MLST specific primers (*27*) and the assembled contigs as input, by using the PubMLST.org database (*28*). For the analysis of *B. canis* genotypes, we compared them to genotypes from the publicly available database MLVA bank (*26*). Of note, the background of reference genotypes is unknown (e.g., import history of the dogs); therefore, these genotypes might not originate from the country in which they were isolated. If >1 isolate was recovered from different materials or time points from a dog in our study, 1 isolate per time point was sequenced with <2 isolates per dog to assess carriage of different genotypes (*29*,*30*).

## Results

Including the first case of canine brucellosis in the Netherlands, 16 suspected cases were notified to NVIC in the study period (Table 1). The reasons for notification are variable: 7 dogs had a seropositive test result at the NRL, 7 dogs had a clinical complaint compatible with *B. canis* infection, and 2 cases had a *B. canis*–seropositive culture (Table 1). Of the 16 dogs, 15 had a history of importation. A total of 10 tested seropositive at the NRL, 4 tested seronegative, and 2 had an inconclusive antibody titer initially but were considered negative during follow-up (retesting after >3 weeks) (Table 1). The 10 seropositive dogs (hereafter referred to as notified seropositive cases) had been imported into the Netherlands 2–32 (median 9) months before notification. Tracking investigations into the 10 notified seropositive cases identified 11 littermates and 13 other contact dogs (Table 1). Of the 11 littermates, 8 were tested by the NRL and all (8/8) were seropositive. Of the 13 contact dogs, 6 were tested by the NRL; 5 were seronegative and 1 had an inconclusive titer (1:50). This dog lived together with notified case dog #12; they had shared an enclosure for 1.5 years with another seronegative contact dog (<1:50). The dog was euthanized because of geriatric health issues and was thus lost to follow-up. Thus, the total number of seropositive cases in this study was 18 (10 notified seropositive cases and 8 littermates) (Table 2).

**Table 1:** Overview of *Brucella canis* notifications and tracking investigations, the Netherlands, November 2016–December 2018*

Notification no.	Notifyingparty	Reason for notification	Clinical diagnosis or complaint	Serologic results (NRL)	Tracking investigation		Case ID
					Litter (positive/tested/ identified), littermates	Contact dogs (positive/tested/identified)	
1	VMDC	*B. canis* positive culture	Discospondylitis	>1:400	NA	0/1/1	1
2	VMDC	Clinical complaint	Epididymitis	˂1:50	NA	NA	
3	VMDC	Clinical complaint	Discospondylitis	>1:400	Litter 1 (2/2/2), 2 littermates	NA	2, 3–4
4	VP	Clinical complaint	Discospondylitis	>1:400	Litter 2 (5/5/8), 5 littermates	NA	5, 6–10
5	NRL	Seropositive	Discospondylitis	1:200	NA	0/3/5	11
6	VP	Clinical complaint	Discospondylitis	1:100	NA	NA	
7	NRL	Seropositive	Discospondylitis	>1:400	NA	1 (inconclusive)/2/3	12
8	NRL	Seropositive	Neck pain	1:100	NA	NA	
9	NRL	Seropositive	Back pain	>1:400	Litter 3 (1/1/1), 1 littermate	0/0/2	13, 14
10	NRL	Seropositive	Behavioral problem	>1:400	NA	0/0/2	15
11	NRL	Seropositive	Discospondylitis	>1:400	NA	NA	16
12	VP	Clinical complaint	Lameness	˂1:50	NA	NA	
13	VMDC	*B. canis* positive culture	Lameness	>1:400	NA	NA	17
14	VP	Clinical complaint	Lameness	˂1:50	NA	NA	
15	VP	Clinical complaint	Epididymitis	˂1:50	NA	NA	
16	NRL	Seropositive	Discospondylitis	>1:400	NA	NA	18

*ID, identification; NA, not applicable; NRL, National Reference Laboratory; VMDC, Veterinary Microbiological Diagnostic Center, Utrecht University; VP, veterinary practitioner.

**Table 2:** Overview of *Brucella canis*–seropositive dogs identified through notifications and tracking investigations, the Netherlands, November 2016–December 2018*

Case	Sex	Birthdate	Litter	Country of origin	Import date	Diagnosis date	Clinical diagnosis or complaint	Diagnostics		
								Serologic result†	Materials cultured	Isolation of *B. canis*
1	Fn	2015 Jul 1		Romania	2016 Feb	2016 Nov	Discospondylitis	>1:400	Urine, blood, intervertebral disc	Intervertebral disc
2	Mn	2015 Aug 13	1	Romania	2016 May	2016 Dec	Discospondylitis	>1:400	Urine, blood, disc	NA
3	Mn	2015 Aug 13	1	Romania	2016 May	2017 Apr		>1:400	Clinical: urine; postmortem: liver, spleen, prostate, Ln. inguinalis, Ln. iliaca	Spleen, Ln. inguinalis, Ln. iliaca
4	Mn	2015 Aug 13	1	Romania	2016 May	2017 Apr		>1:400	Urine	NA
5	Mn	2015 Oct 1	2	Bulgaria	2016 Feb	2016 Dec	Discospondylitis	>1:400	Urine, blood	Blood
6	Fn	2015 Oct 1	2	Bulgaria	2016 Feb	2016 Dec	Discospondylitis	>1:400	Urine, blood, synovial fluid	NA
7	Fn	2015 Oct 1	2	Bulgaria	2016 Feb	2017 Jan		1:200	NA	NA
8	Fn	2015 Oct 1	2	Bulgaria	2016 Feb	2017 Jan	Discospondylitis	>1:400	Urine, blood	Urine, blood
9	Fn	2015 Oct 1	2	Bulgaria	2016 Feb	2017 Jan	Discospondylitis	>1:400	Clinical: urine; postmortem: blood, liver, spleen, kidney, lung, Ln. iliaca, intervertebral discs (3)	Urine, spleen, lung, Ln. iliaca
10	Mn	2015 Oct 1	2	Bulgaria	2016 Feb	2017 Jan	Discospondylitis	>1:400	Urine, blood	Urine, blood
11	Fn	2008 Jul 19		Romania	2016 Aug	2017 Mar	Discospondylitis	1:200	Urine	NA
12	F	2015 Aug 23		Croatia	2015 Dec	2017 Jun	Discospondylitis	>1:400	Urine	Urine
13	F	2017 Feb 15	3	Romania	2017 Jun	2017 Oct	Back pain	>1:400	Urine, blood	Blood
14	Mn	2017 Feb 15	3	Romania	2017 Jun	2017 Nov	Neck pain	>1:400	Urine, blood	Urine, blood
15	Mn	2014 Dec 1		Bulgaria	2015 Oct	2017 Nov	Behavioral problem	>1:400	Urine, blood	Blood
16	M	2017 Jul 16		Bulgaria	2018 Feb	2018 Apr	Discospondylitis	>1:400	Urine	NA
17	Fn	2014 Dec 1		Bulgaria	2015 Sep	2018 May	Lameness	>1:400	Clinical: synovial fluid; postmortem: urine, blood, lung, kidney, spleen, liver, knee joints (including synovial fluids), vagina, Lnn. iliaca, Lnn. popliteus, Ln. mesenterialis	Synovial fluid (clinical sample)
18	Mn	2017		Bulgaria	Unknown	2018 Dec	Discospondylitis	>1:400	NA	NA

*Fn, female neutered; Ln, lymph node; Lnn, lymph nodes; Mn, male neutered; NA, not applicable.  †By 2-Mercapto-ethanol serum agglutination test.

Of these 18 dogs, 14 (78%) had musculoskeletal disease with such clinical signs as lameness and neck or back pain; discospondylitis was diagnosed in 11. Information regarding onset of clinical signs was available for 7 of these 13 dogs and occurred 0–3 months after import. One dog had a behavioral problem and 3, all littermates identified through tracking investigations, showed no clinical signs.

All dogs were mixed-breed rescue dogs imported from Romania (n = 7), Bulgaria (n = 10) and Croatia (n = 1). Among them were 9 female dogs, of which 7 were neutered, and 9 male dogs, of which 8 were neutered.

We collected blood, urine, or samples from the infection site from 16 of 18 seropositive dogs for culture; 10 dogs tested positive on these clinical samples (Table 2). Three dogs (nos. 3, 9, and 17) were euthanized because of deteriorating clinical symptoms linked to brucellosis; postmortem examination and cultures revealed growth of *B. canis* in collected tissue samples in 2 of 3 dogs (Table 2). This brings the total number of culture-positive cases in this study to 11 (10 from clinical samples and 1 exclusively from postmortem tissue samples) (Table 2). We cultured isolates from blood (6 samples), urine (5 samples), lymph nodes (3 samples), spleen (2 samples), lung (1 sample), synovial fluid (1 sample), and intervertebral disc (1 sample) (Table 2).

Genotyping was performed for 14 isolates; from 3 dogs, >1 isolate was recovered at different time points. Genotyping of isolates confirmed a close relation between isolates from the same litter (Figure). Isolates from dogs imported from Bulgaria show high similarity. Isolates from litter 3 imported from Romania show high similarity, and the isolate from dog#3 clusters with a reference strain from Romania. Only the isolate from dog 1 does not cluster with any other (reference) strain from Romania. On the basis of in silico MLVA-16 analyses, the 2 isolates from dog 5 (samples taken with a 12-month interval) showed no difference in loci. The 2 isolates from dog 9 (3-month interval) showed 1 locus difference (MLVA Bruce16: first isolate 7 repeats, second 8 repeats). The 2 isolates from dog 8 (6-month interval) showed 2 loci difference (MLVA Bruce09: first isolate 7 repeats, second 6 repeats; Bruce16: first isolate 8 repeats, second 9 repeats).

**Figure:**
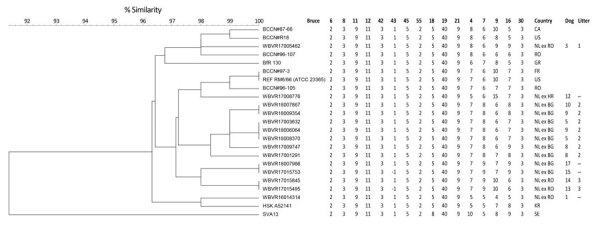
Multilocus variable-number tandem-repeat analysis 16 clustering analysis of 14 *Brucella canis* isolates (indicated with prefix WBVR, chronologically numbered) in conjunction with reference strains. Numbers indicate repeats per locus for different *B. canis* isolates; when unknown, the number is −1. BG, Bulgaria; CA, Canada; ex, dog imported from; FR, France; GR, Greece; HR, Croatia; KR, South Korea; NL, the Netherlands; REF, reference strain; RO, Romania; SE, Sweden; US, United States; WBVR, Wageningen Bioveterinary Research.

## Discussion

Brucellosis in dogs is not notifiable to the OIE or the European Union; therefore, prevalence data on canine brucellosis in different countries are scarce. Literature does confirm occurrence of *B. canis* in stray dogs in Bulgaria (*31*,*32*) and reports bacterial isolates from dogs in Romania (*16*,*33*). Buhmann et al. give an overview of test results for *B. canis* on the basis of data from a large laboratory in Europe receiving samples from 20 different countries in Europe. However, the background of the dogs (i.e., country of origin) is unknown, which makes it difficult to assess the risk of importing dogs from specific countries of origin (*13*).

The Netherlands imports an estimated 21,000 dogs legally per year (unpublished report, NVWA, 2018). According to the TRAde Control and Expert System (TRACES, https://webgate.ec.europa.eu/sanco/traces), a mean of 3,433 (range 2,925–3,950) dogs per year were imported from Romania, 724 (range 557–986) dogs per year from Bulgaria, and 20 (range 9–34) dogs per year from Croatia for the period 2015–2018. This case series underlines the risk of importing dogs from countries to which *B. canis* is endemic. Because *B. canis* was never isolated in the Netherlands before and most dogs showed clinical signs of infection shortly after arrival, all cases are considered import cases. This supposition is supported by the analysis of the genotypes, which showed clustering of isolates within litter and country. Minor differences between genotypes (1 or 2 loci) were seen in isolates from the same dog or litter, which might be explained by coinfection or within-host evolution (*29*,*30*).

The clustering of isolates within a litter confirms vertical transmission of *B. canis.* The most common transmission route of *B. canis* is venereal. Most dogs in our study (15/18) were neutered, which reduced the risk for transmission through genital secretions. Urinary shedding has been implicated as a possible transmission route for dogs cohabiting with male dogs (*4*,*5*). Bacteriuria has been demonstrated in both sexes; however, female dogs appear to shed a lower number of bacteria per milliliter (*5*). Serikawa et al. demonstrated up to 10^6^ bacteria/mL urine in male dogs, which supports potential transmission of *B. canis* through urinary shedding (*4*). To our knowledge, all studies on urinary shedding have been conducted with intact animals. Shedding by neutered dogs is believed to be less likely (*34*), but evidence to confirm this does not exist. In our case series, shedding of *B. canis* in urine was found in 4/13 (31%) neutered dogs and 1/3 (33%) intact dogs, indicating that shedding by neutered dogs does occur and should be taken into account. Further research into the number of bacteria shed through urine of neutered dogs infected with *B. canis* is warranted to assess the risk for transmission to other animals or humans.

The diagnosis of a *B. canis* infection in dogs is hampered by subclinical disease and nonspecific clinical signs. In addition, both serologic testing and bacterial isolation have their limitations because of the nature of the disease (*34*). To avoid spread of canine brucellosis, dogs should be tested before international movement (*7*). This process should involve a combination of tests at different times (*34*). However, freedom of trade between European Union member states hampers the unilateral introduction of mandatory control measures.

The zoonotic risk associated with the dogs infected with *B. canis* in our case series relates mostly to owners, veterinary personnel, and laboratory technicians. Laboratory personnel were put at risk by the positive cultures of dogs 1 and 17, because routine diagnostic procedures were done under BSL-2 conditions, whereas BSL-3 is mandatory for all *Brucella* spp. The risk level of the technicians involved was assessed by medical microbiologists of the Municipal Health Service in line with national guidelines (*35*). To our knowledge, no human infections were linked to the cases documented in this study. However, with the ongoing import of dogs from areas to which *B. canis* is endemic, aspiring dog owners, veterinary personnel, and laboratory technicians will continue to be at risk. Without mandatory testing or control measures, the competent authority in the Netherlands can only inform owners on the poor prognosis and the zoonotic risk and discuss the options of euthanasia or neutering of sexually intact dogs.

In conclusion, this case series proves introduction of *B. canis* in a country to which it is not endemic through import of infected dogs from *B. canis*–endemic areas, posing a threat to the naive autochthonous dog population and to humans. The extent of this threat is hard to estimate because of lack of prevalence data and mandatory testing combined with challenges in diagnosing the infection. Furthermore, the case series indicates that shedding of *B. canis* in urine by neutered dogs occurs and should be considered when assessing the risk for transmission.
